# Interplay between evanescence and disorder in deep subwavelength photonic structures

**DOI:** 10.1038/ncomms12927

**Published:** 2016-10-06

**Authors:** Hanan Herzig Sheinfux, Ido Kaminer, Azriel Z. Genack, Mordechai Segev

**Affiliations:** 1 Technion, Israel Institute of Technology, Haifa 32000, Israel; 2 Department of Physics, Massachusetts Institute of Technology, Cambridge, Massachusetts 02139, USA; 3 Physics Department, Queens College and Graduate Center of CUNY, Flushing, New York 11367, USA

## Abstract

Deep subwavelength features are expected to have minimal impact on wave transport. Here we show that in contrast to this common understanding, disorder can have a dramatic effect in a one-dimensional disordered optical system with spatial features a thousand times smaller than the wavelength. We examine a unique regime of Anderson localization where the localization length is shown to scale linearly with the wavelength instead of diverging, because of the role of evanescent waves. In addition, we demonstrate an unusual order of magnitude enhancement of transmission induced due to localization. These results are described for electromagnetic waves, but are directly relevant to other wave systems such as electrons in multi-quantum-well structures.

## Abstract (web summary)

Features much smaller than the wavelength are not expected to have a significant impact on the transport of a wave. Here, the authors show that Anderson localization can dominate light transport in a one-dimensional disordered system, even when the disordered features are a thousand times smaller than the wavelength.

## Graphical abstract

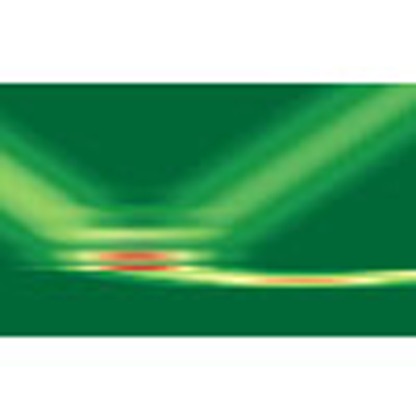

Localization due to disorder was originally proposed by Anderson^1^ in 1958 in the context of the metal insulator transition in solid-state physics. Over the years, it has been shown that localization phenomena are fundamental and ubiquitous^2^,^3^, manifested in diverse realms of physics—from photonics^4^,^5^,^6^,^7^,^8^,^9^,^10^,^11^,^12^ and cold atoms^13^,^14^ to sound waves^15^, surface plasmons^16^ and various mesoscopic systems^17^. Anderson localization occurs as a result of constructive interference of scattered waves returning to their origin point in the medium along time-reversed paths. Being an interference effect, localization relies on phase accumulated between scattering events. Its impact is therefore expected to decrease when the distance between the scatterers becomes much smaller than the wavelength, *λ*, since the accumulated phase is then much smaller than 2*π*. Indeed, when *d*<<*λ*, the typical length scale of localization, *ξ*, diverges rapidly^18^,^19^,^20^. Accordingly, a subwavelength disorder with 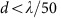 is expected to induce very weak localization, which is usually physically negligible. Localization in this case is characterized by very long *ξ*, which diverges rapidly as *λ*^2^, and disorder only plays a role if the disordered structure is exceedingly large. This insensitivity to extreme subwavelength detail is, of course, not unique to Anderson localization or disordered structures; rather, any potential with such extreme subwavelength features is, generally and intuitively, expected to have little to no influence on wave transport.

Importantly, propagating waves are not the only type of waves scattered by disorder. Evanescent (exponentially decaying) waves are also scattered, and while evanescent waves naturally do not accumulate phase through propagation, they can accumulate a phase as they scatter via the Goos–Hanchen phase shift. Notably, evanescent waves acquire this phase shift ‘immediately’ upon reflection from an interface (or transmission through it), as opposed to a phase shift proportional to the propagation distance. This raises a fundamental question: can localization be induced by multiple scattering of evanescent waves? And if so, can this type of localization overcome the diffraction limit and persist in the deeply subwavelength (*d*∼*λ*/50) and extreme subwavelength (*d*∼*λ*/1,000) regimes?

Perhaps, the simplest setting in which localization can occur is the specific case of electromagnetic (EM) waves in multilayer structures. Localization in disordered multilayers was proposed as early as 1985 (refs 19, 21, 22) and later observed experimentally^23^,^24^, but almost all of the current literature deals exclusively with the localization of propagating waves, with no mention to evanescent waves. An important exception was found in refs 21, 22, which examined a multilayer stack with thick (wavelength-scale) layers and permittivities that are distributed randomly with a perturbatively small variance. When light is incident on the multilayer exactly at the (mean) critical angle for total internal reflection, it was shown^21^,^22^ that transmission decays exponentially with the number of layers. However, the origin of this decay—whether it was Anderson localization or simple evanescent decay—was not determined. In fact, an important later work^25^ showed that if the waves are evanescent in a large number of layers, the reduction in transmission has ‘nothing to do with localization’ (as quoted from ref. 25). In all, before our work, the possibility of Anderson localization in a structure composed entirely of deep subwavelength layers has not been considered.

In the following, we demonstrate that Anderson localization can be induced by extreme subwavelength disorder and driven by evanescent waves. We show that multiple Goos–Hanchen phase shifts of scattered evanescent waves can induce a potent Anderson localization regime, despite the subwavelength scale of the structural disorder. As a concrete example, we study the transport of EM waves through a stack of subwavelength dielectric layers, which alternatingly support propagating and evanescent waves. In the presence of disorder, transmission through the stack is reduced and exponentially localized modes appear. We show that the transport regime which ensues can indeed be characterized as Anderson localization, even for *d*∼*λ*/1,000 and that the localization length is as short as a few wavelengths. Moreover, we prove, analytically, that the localization length scales linearly with the optical wavelength instead of diverging parabolically. In addition, we demonstrate that disorder-induced localization can enhance transmission by orders of magnitude through some realizations of disordered finite multilayer stacks. The modes responsible for enhanced transmission are shown to possess untypically high Q-factors, up to the 10^6^ range, which exist even in relatively thin (*L*∼2–8*λ*) structures. Combining the inherent sensitivity of our regime with the presence of high-Q modes may result in major sensing applications, where optical wavelengths are used to resolve nanometric details in a multilayer structure.

## Results

### Our model and the role of evanescent waves

**Figure 1:**
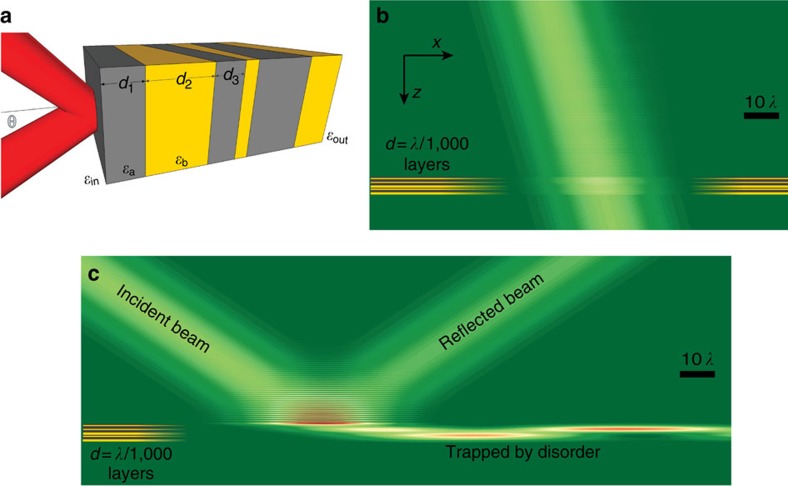
Physical setting and simulated propagation of light in the multilayer stack with extremely deep subwavelength disorder. (**a**) Schematics of a disordered multilayer sample with *N*=3 pairs. (**b**) A TE-polarized 2D Gaussian beam incident at *θ*=20^°^ on a disordered stack of *N*=2,500 layer pairs. The beam goes through the structure unaffected by the disorder since the layers are 10 nm thick on average—exceedingly thin relative to the *λ*=10 μm wavelength of the beam—and since the incidence angle is far from the critical angle for total internal reflection. (**c**) The same beam and structure as in **a**, but for incidence at *θ*=60^°^. For this angle of incidence, at the edge of the GHL regime, the beam is completely reflected by the presence of deep subwavelength disorder. Notably, a portion of the beam is coupled into a mode localized in the multilayer, and advances through the structure for a considerable distance. The yellow–grey stripes on the bottom-left hand side of **b**,**c** represent the location of the multilayer; the actual layers are 500 times thinner than in the illustration.

Consider a one-dimensional (1D) structure made up of 2*N* dielectric layers, illuminated by a continuous-wave laser (CW) source at an angle of incidence *θ*, wavelength *λ* and TE polarization, as shown in Fig. 1a. The multilayer stack is surrounded by a homogenous medium with permittivity *ε*_ext_=4, and the permittivity of the layers alternates between the low and high values *ε*_L_=1 and *ε*_H_=5. Disorder is introduced by drawing the layer thickness randomly from a uniform distribution between 2 and 18 nm. This choice of parameters is quite arbitrary and the results henceforth described would be achieved for other choices, as long as *d*<<*λ* and *ε*_ext_,*ε*_h_ are sufficiently greater than *ε*_L_ (see ref. 26). Another physical requirement is that the thickness of the layers will be sufficiently large for the dielectric permittivity to be well defined and close to its bulk value. An experimental effort will certainly be affected by such deviations, and, while the precise minimum thickness depends on the fabrication method and materials, a minimum thickness of 2 nm seems to be a reasonable estimate for most dielectrics^27^. We emphasize that the disorder could also be introduced into the multilayer in a variety of other ways. For example, we also studied other realizations of disorder, such as randomly reshuffling the layers or randomizing the layer permittivities, and found that the main results we describe remain valid. We note that, for *d*∼*λ* or larger, the evanescent decay inside the low-permittivity layers is so significant that waves do not penetrate into the structure and therefore do not experience the disorder. We also note that, since *d*<<*λ*, analysis in terms of Bloch states is not helpful.

At first glance, it might appear that transport in this deeply subwavelength-layered system could be treated using effective medium theory, in which the effects of individual layers would be smoothed, so that the wave responds only to an averaged structure (see refs 28, 29). However, it is not always possible to apply the effective medium approach. For example, we have recently shown that transmission through a periodic multilayer stack (free of disorder) can depend on structural features such as the order and exact number of the layers^26^, which are neglected in the conventional effective medium approach. In such cases, it is necessary to use the full solution of the wave equations with a method such as the transfer matrix method^30^,^31^ employed in this article. Nevertheless, we find that some concepts from effective medium theory still apply. Namely, we find that the effective medium permittivity *ε*_eff_ (in our case is 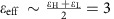) and especially the critical angle for total internal reflection, 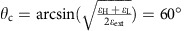 (for a wave propagating from an external medium with *ε*_ext_ to a homogeneous medium with *ε*_eff_), are excellent predictors of whether the overall field behaves like an evanescent or propagating wave within the medium.

We can identify four regimes of operation in different ranges of *θ*:

0≤*θ*<*θ*_L_: where for our chosen parameters 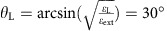. In this regime, waves propagate with real wavevectors in both layers. This is the regime of conventional Anderson localization. The length scale characterizing localization diverges as *ξ∼λ*^2^ or faster^18^,^19^,^20^, so that localization due to a deep subwavelength disorder is generally a weak effect.

*θ*_L_<*θ*<*θ*_c_: with *θ*_c_=60°. In this regime, waves in the *ε*_L_ layers are evanescent, whereas waves in the *ε*_H_ layers remain propagating waves. Crucially, the evanescent nature of the waves in the *ε*_L_ layers does not necessarily imply exponentially reduced transmission through the disordered multilayer structure. In a perfectly periodic multilayer of this kind, the transmission is a periodic function of the total thickness, and even full transmission can be obtained in arbitrarily thick multilayers, in spite of evanescence^26^. However when the layers are disordered, we find that the Goos–Hanchen phase shift (that is, the phase of Fresnel reflection\transmission coefficients) plays an increasingly important role, and that short-range localization can take place even if the disorder is deep subwavelength. Accordingly, we refer to this regime as the Goos–Hanchen localization (GHL) regime, which reflects the mechanism underlying this new regime of localization.

*θ*_c_<*θ*<90°: In this regime, as in the GHL regime, the wave is propagating in the high-permittivity layers and is evanescent in the low-permittivity layers. In the effective medium description, the wave in this regime is evanescent and displays an exponential decay in transmission even in a periodic structure without disorder^26^. In a fully periodic multilayer structure, the rate of amplitude decay is close to the rate of decay in the effective medium model, 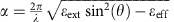. In contradistinction, the presence of disorder endows the structure with a complex behaviour because of the interplay between the effective evanescent decay and Anderson localization, which leads to enhanced transmission and unusual mode shapes, which will be described later. We call this angular range the effective medium evanescence (EME) regime because the overall transmission decays exponentially.

Depending on the surrounding permittivity, a fourth regime might also exist in which the waves are evanescent in *both* layers. For the parameters we consider here, *ε*_H_>*ε*_ext_; hence, this does not exist for any (real) angle. However, in general, we find that evanescence in this regime is so dominant that the effects of disorder are vanishingly small.

### The GHL regime

Consider first the regime of conventional localization, with *θ*<*θ*_L_. We calculate the field distribution of a two-dimensional (2D) Gaussian beam incident at *θ*=20° on a single (arbitrarily chosen) disordered multilayer, with *N*∼2,500 layer pairs, using transfer matrix formalism. We begin with the extreme case of *λ*=10 μm, relative to a *d*=10 nm layer thickness, corresponding to a localization length scale of several millimetres. As shown in Fig. 1b, the effect of disorder in this case is weak—almost all of the light is transmitted. The small amount reflected, evident from the faint interference pattern, is comparable to the reflection predicted by effective medium theory because of the permittivity difference between *ε*_eff_ and *ε*_ext_. For thicker structures, some effects of disorder can appear, but full localization is only expected in implausible structures with more than *N*∼10^5^ layer pairs.

In contrast to the above case of near-normal incidence, when the same beam is incident at *θ*_c_=60° on the same structure, it is completely reflected by the disorder. As shown in Fig. 1c, almost no power is transmitted through the multilayer, even though the overall thickness of the stack is only ∼5*λ*. Figure 1c also demonstrates that a portion of the beam is ‘trapped’ inside the multilayer and travels laterally inside the multilayer for an appreciable distance—a clear signature of the presence of strongly localized Anderson modes. We will return to analyse the modes and transverse behaviour later on. This lateral shift is reminiscent of the Goos–Hanchen effect in total internal reflection, but unlike the Goos–Hanchen effect, the transverse shift here is frequency-dependent and is extremely large. Additional examples, for other realizations of disorder, are shown in Supplementary Note 3. Ultimately, Fig. 1c (and the additional examples in Supplementary Fig. 3) demonstrates that the extreme subwavelength features of our structure completely dominate the transport of light.

### Scaling of the localization length with the wavelength

From this point onwards, we simplify the setting by treating the incident beam as a plane wave of infinite extent and singularly defined angle of incidence instead of a Gaussian beam. Specifically, we calculate the localization length, defined as *ξ*=−〈ln*T*〉/*L* (refs 24, 25), for a plane wave incident at some angle from an external medium with *ε*_ext_ into the multilayer. Here the averaging denoted by <…> is performed over a large ensemble of realizations of the disorder, *T* is the transmission through a specific realization and *L* is the overall thickness of the structure. Our calculations are performed for *L*>>*ξ*.

**Figure 2:**
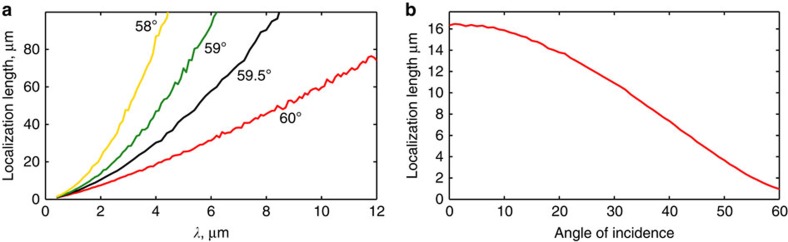
Characteristics of localization by deep subwavelength disorder. (**a**) Localization length as a function of wavelength *λ* for incidence at *θ*_c_=60^°^ and several other angles of incidence. As explained in the text, this plot is drawn for wavelengths, which are much larger than the layer thickness. (**b**) Localization length as a function of *θ*, the angle of incidence for *λ*=0.5 μm.

For conventional Anderson localization and for most of the GHL regime, the localization length diverges as the square of the wavelength, *ξ*∼*λ*^2^. However, for waves incident at an angle close to *θ*_c_, we find (Fig. 2a) that *ξ*∼6*λ*. The localization length remains very short even for extreme subwavelength disorder. For planewaves, incident at any *θ*<*θ*_c_, *ξ* eventually diverges for asymptotically large wavelengths. So the angular bandwidth over which localization plays a dominant role in transport does diminish with increasing wavelengths. This raises an immediate question regarding the feasibility of observing such effects experimentally, for very large *λ*, because any physical beam (a wavepacket) has a non-zero angular bandwidth, unlike the ideal plane wave considered here. This therefore presents a physical limit preventing localization from being relevant to infinitely large wavelengths (or, equivalently, to infinitely small disordered features). However, as evident in Fig. 1c, such effects should be physically meaningful even for *d*∼*λ*/1,000, far beyond what is usually considered possible. The angular bandwidth with *d*∼*λ*/1,000 is still sufficient to dictate the transmission of realistic beams with finite width. These results suggest a new approach for optical sensing of deep subwavelength structural features, enabled by a completely different mechanism than previously suggested super-resolution techniques.

To see the transition from ‘ordinary’ Anderson localization to the GHL regime, we set *λ*=1 μm and calculate *ξ* as a function of *θ*. We see in Fig. 2b that *ξ* decreases as *θ* increases, indicating stronger localization. In the range 0<*θ*≤*θ*_*L*_=30° this decrease is intuitive—larger angles of incidence imply larger Fresnel reflection coefficients at each interface, and therefore stronger scattering and shorter localization lengths.

The transition into the GHL regime, as shown in Fig. 2b, is smooth. This is surprising since phase accumulates differently for *θ*<30° and 30°<*θ*<60°. Beyond *θ*=30°, the wave alternates between being evanescent and propagating. Accordingly, the Fresnel reflection coefficients are complex with unity magnitude. Phase in this case is accumulated by Fresnel reflections from the interfaces between the layers, as well as regular propagation. Moreover, the Fresnel transmission coefficients are actually larger than unity (but power is preserved and the time-averaged Poynting vector is constant^26^). However, no distinct kink or sharp transition is seen in Fig. 2b around *θ*_*L*_=30°; rather, *ξ* continues to drop monotonically until it reaches its minimum value at *θ*_c_=60° at the exceedingly small value of *ξ*=1.1 μm. This is comparable to the shortest localization lengths found in near-normal incidence for disordered multilayers with *λ*/4 average layer thicknesses^32^, which are commonly believed to yield the strongest localization lengths. In principle, we could continue to even larger angles of incidence. However, for *θ*>*θ*_c_ we are at the EME regime and transmission drops exponentially because of evanescence—even without disorder. In this case, 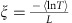 describes the effect of both disorder-induced-localization and EME. Usually, *ξ* also represents the typical longitudinal length scale of the localized modes, but in the EME regime we find that this is not necessarily true and separating the roles of localization and evanescence can be a major challenge.

### Analytical derivation of the localization length scaling

To better understand the origin of these results, we investigate the GHL regime analytically. The full proof is found in Supplementary Note 4, while here we provide only the principal arguments and insights they produce. Specifically, we focus on a wave incident at *θ*=*θ*_c_, and show that *ξ* indeed is linear with the wavelength, going as 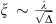, with 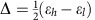, in accordance with the numerical results in Fig. 2a and in Supplementary Fig. 11.

The approach we consider here utilizes the transfer matrix formalism, which yields an exact solution of Maxwell's equations in 1D. From the transfer matrices, we find the average rate of transmission decay using the Hamiltonian mapping method, which maps the transfer matrix problem to a trajectory in the *E*_*y*_,*B*_*x*_ plane (the *y* and *x* components of the electric and magnetic fields, respectively), as a function of the number of layers. Alternative methods include transfer matrix theory^33^, Green function-based approaches^34^ and mapping the problem to the Fokker–Planck equation^35^,^36^. These alternative methods are very rigorous and accurate, but are less suited to the case at hand than the Hamiltonian mapping technique, which we use here, based on the methods of ref. 37.

The first step we take is to consider the evolution of the system under the influence of the unperturbed Hamiltonian, in which disorder is absent. Away from the critical angle, the phase accumulated per bilayer is *γ*(λ>>*d*)∼*k*_*z,h*_*d*. However, at critical incidence, *θ*=*θ*_c_, we show in Supplementary Note 4 that phase accumulates much more slowly 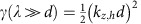. The trajectory in the *E*_*y*_,*B*_*x*_ plane at critical incidence maps into an elliptical orbit, extremely narrow in the *B*_*x*_ axis. But in the *λ*>>*d* limit, the amplitude of the disorder-induced fluctuations in *B*_*x*_ can be much larger than the unperturbed *B*_*x*_. Consequently, the naive perturbation approach fails. Instead, we make the ansatz that in the presence of disorder the amplitude *B*_*x*_ of the basic trajectory is *λ*/*d* larger than in the naive effective medium case. The intuition for this ansatz comes from calculating the characteristic size of *B*_*x*_ to the lowest order in *λ*/*d* in the presence of minimal disorder. Following this first stage, we include additional disorder as a perturbation upon this new basic state. This ansatz is supported by numerical calculations (see Supplementary Note 4). Using this assumption, we find the average rate of change in the radius of the *E*_*y*_,*B*_*x*_ trajectories and deduce the scaling relation 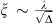. Importantly, this derivation provides significant insight into the origins of the unusual linear scaling relation—it shows that for critical incidence, and only for critical incidence, the importance of effective medium decays with increasing *λ* much faster than the importance of disorder-induced perturbations.

Another interesting insight gained from the analytical description relates to the influence of structural correlations to the strength of localization. It is well known that, for certain types of disorder, unusual effects such as Fano resonances^35^ can appear, but introducing correlations often destroys localization^38^. However, some types of correlations lead to more severe delocalization than others. In our case we find that severe delocalization occurs if both layers have the same random thickness (*d*_2*n*_=*d*_2*n*+1_). For this case, we show in Supplementary Note 5 that the localization length scales exceedingly fast, as *ξ*∼*φ*^4^. This relation is the same as in ref. 37, but the distinction between correlated and uncorrelated disorder in our case becomes quite astounding. ‘Switching on’ correlations, which are correlations on the very deep subwavelength scale, can move the localization length from *ξ*∼5*λ* in the uncorrelated case for *λ*/1,000 to an excessively long *ξ*∼10^6^*λ*. This is further evidence of the influence deep subwavelength features can have on transport in this regime.

### Characterizing Anderson localization

Before proceeding, it is crucial to explicitly address the question of Anderson localization. So far, we have demonstrated strong exponential decay in transmission through a disordered multilayer, but did not explicitly show that this decay stems from randomness and disorder in the multilayer. In particular, the transmission decay we have shown (Figs 1c and 2) could be interpreted as a direct consequence of the evanescent decay occurring in half of the layers in the structure, regardless of the disorder. It is therefore essential to test whether our results are truly induced by disorder and can be identified as Anderson localization.

We demonstrate that localization is induced by disorder in three ways (see also additional the discussion in Supplementary Note 2):

First, we find that in absence of disorder and for *θ*<*θ*_c_, we see no exponential decay in transmission with increasing sample thickness. In fact, full transmission can be obtained through a periodic multilayer with an arbitrarily large number of layers. Only when disorder is added, do we see the exponential decay in transmission and all of the other effects we describe. Therefore, disorder is essential for the decay in transmission we observe and the other effects associated with it. Evanescence in the layers or the vicinity of *θ*_c_ is not sufficient to induce localization.

Second, we examine the statistical distribution of transmission in our regime and show it is a log-normal distribution and also this distribution obeys single-parameter scaling (see Supplementary Fig. 1). These are both well-known characteristics of Anderson localization in 1D (refs 17, 39, 40).

Third, we show that the field distribution inside the layered structure does not decay monotonically (as is the case for simple evanescent decay). Instead, the field is a sum of fields in distinct transmission modes, which are strongly localized even for *d*∼*λ*/1,000, as shown in Supplementary Fig. 2. As expected for Anderson localization, these modes have a Lorentzian frequency dependence and are characterized by a length scale similar to *ξ*.

We can also better understand the transverse propagation seen in Fig. 1c in this modal approach. The trapped energy is energy-coupled in high-Q modes inside the sample. We find that these trapped modes often exist and have surprisingly high-Q factors, reaching a maximum of several times 10^4^ (for the given configuration parameters). Moreover, some high-Q mode is almost always found in the vicinity of the critical angle and there is, on average, a mode with Q-factor of ∼200 that can play a role for beams incident at *θ*_c_. Accordingly, these modes trap part of the beam's energy for a significant length, and it is relatively easy to find realizations in which the beams travel for very extended lengths inside the structure.

### Enhanced transmission at incidence above the critical angle

**Figure 3:**
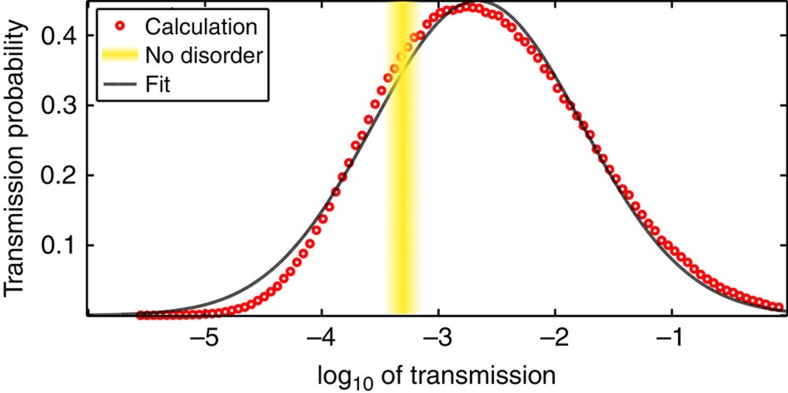
Probability density of transmission in the EME regime. Transmission probability density for an *N*=180 structure (red circles), generated from an ensemble of 1 million realizations of the disorder. The distribution is compared against a Gaussian fit (black curve) and against the transmission of the disorder-free structure *T*≃10^−3.3^ (yellow stripe).

Next, we consider the EME regime in which waves are incident at *θ*>*θ*_c_ and transmission is expected to decay exponentially even in a disorder-free system. As before, the modes of the system are strongly localized with Lorentzian spectral lines. However, the probability distribution of transmission (shown in Fig. 3 for *θ*=62°>*θ*_c_) differs slightly from a log-normal distribution and the width of the distribution is roughly half that expected from the single-parameter scaling hypothesis. This indicates that propagation is modified fundamentally by the coexistence of localization and evanescence in this regime. It is intriguing to compare the transmission in Fig. 3 with transmission through a periodic (disorder-free) structure. For some realizations of disorder, the transmission through the disordered structure is lower than in the periodic one, but in most of the realizations, adding disorder enhances the transmission dramatically. Notably, in ∼4% of the realizations, transmission increases from *T*∼0.0005 in the periodic structure to *T*>0.1 in the disordered sample.

This finding contrasts with disorder-enhanced transport in periodic^41^ or quasi-periodic structures^11^. In those studies, the confinement that restricts the transmission results from the order in the structure, and the enhancement of transmission is a result of the disruption in the periodic structure. Unlike those cases, the confinement mechanism here, evanescence, arises in the effective medium description. It depends only on the averaged permittivity, which does not change in the presence of disorder and, in addition, occurs over a broad bandwidth. Therefore, our results indicate that it is really localization that is counterintuitively responsible for enhancing transmission. Our findings are also related to the prediction of disorder-enhanced quantum tunnelling^42^. However, the enhancement predicted by ref. 42 and other works following ref. 42 is a mild effect, which is not expected to raise the transmission to a significant level. For example, in our case, it is similar to the increase seen in Fig. 3 from log_10_*T*=−3.3 in the disorder-free structure to 〈log_10_*T*〉=−2.8, which, while higher, is still a very low transmission. What we show here, on the other hand, is that for some sample realizations, disorder-induced localization elevates the system from being extremely reflective to transmitting an appreciable part of the energy.

### Localized modes in the EME regime

**Figure 4:**
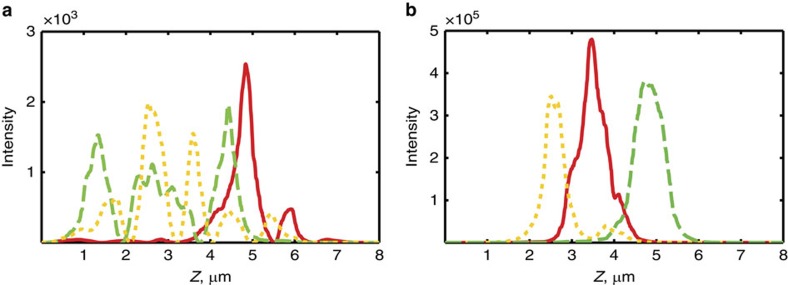
Typical modes above and below *θ*_c_. (**a**) Three localized modes for *θ*=58^°^ and *λ*=1 μm. These modes were found by considering realizations with high transmission (in an ensemble of 10,000 realizations) and calculating the field distribution. (**b**) Same, for *θ*=62^°^.

These results reflect the interplay between evanescence and disorder (where by evanescence we are referring here to EME). While both Anderson localization and evanescence lead to the exponential decay of average transmission, the mechanism for the decay is fundamentally different. Namely, the evanescent field in a uniform or periodic sample decays monotonically into the sample. On the other hand, in disorder-induced localization, the field profile is more complex, and the intensity inside the structure can be much higher than at the input, especially near resonance with localized modes. This leads to increased transmission in some realizations. The profile of the localized modes is illustrated in Fig. 4. This figure shows the energy-density distribution in three high-transmission realizations of disorder in the GHL and EME regimes. In the EME regime (Fig. 4b), the modes are centred inside the structure, have a high Q-factor and exhibit an overall exponential decay away from their peak, with a length scale close to the exponential decay length in the corresponding disorder-free periodic structure. In our deep subwavelength structure, the role of these modes in enhancing transmission is similar to localized modes in the bandgap of a disordered photonic crystal.

A salient feature of the modes shown in Fig. 4b is that they exhibit a single peak—they rise and fall without large oscillations in intensity and at the same time are strongly localized with Q-factors that can exceed 10^5^, even in a sample only a few wavelengths thick. This behaviour is very different from what is typical in localized modes—these exhibit many oscillations (and nodes), superimposed on top of an envelope, which decays exponentially in a distance roughly equal to the localization length. For example, compare Fig. 4b with Fig. 4a, for which the angle of incidence is changed from *θ*=62°>*θ*_c_ (EME regime) to *θ*=58°<*θ*_c_ (GHL regime) in a specific random realization of the disorder. This slight change in *θ* translates to a completely different landscape of modes—unlike in Fig. 4b, the localized functions in Fig. 4a possess multiple peaks and nodes and lower Q-factors. In other words, the structure of the resonant transmission modes in our deep subwavelength multilayer stack in GHL and EME regimes differ dramatically. For *θ<θ*_c_ the modes behave like traditional Anderson localized modes, even though in half of the layers the waves are evanescent. When *θ* approaches and exceeds *θ*_c_, however, we start seeing localized modes rise and fall without oscillations and have high Q-factors.

**Figure 5:**
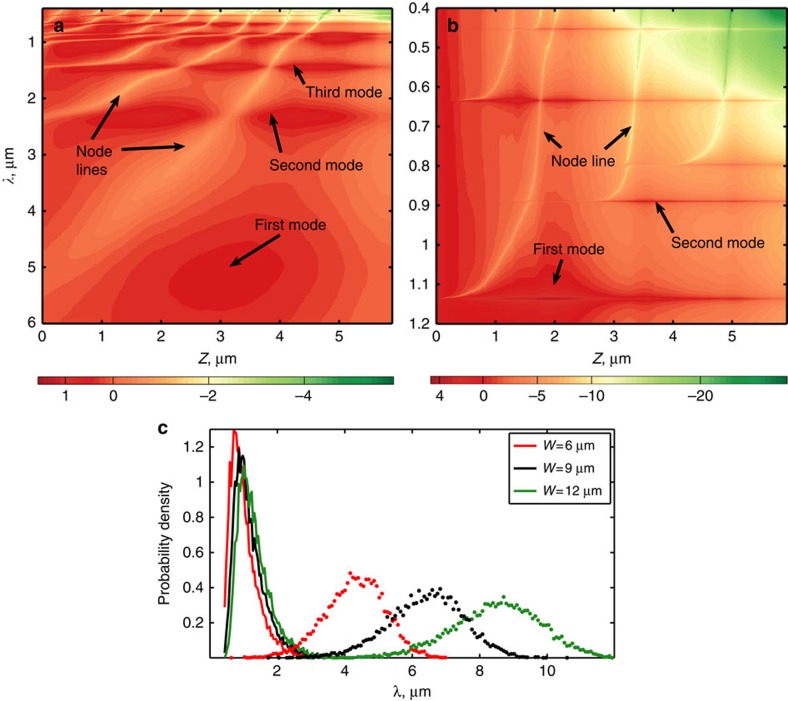
Mapping of the first transmission modes above and below *θ*_*c*_. (**a**) Distribution of log_10_ of the intensity, as a function of *z* (the location inside the sample) and wavelength *λ*. The colourmap is normalized. This plot represents a specific (arbitrarily chosen) structure with *N*=300 layer pairs illuminated at *θ*=58^°^. Note that the colourbar immediately below is logarithmic (powers of 10). (**b**) Same as (**a**), for illumination at *θ*=62^°^. (**c**) Probability distribution of the wavelength of the first mode for two angles of incidence *θ*=58^°^ (dotted line) and *θ*=62^°^ (solid line).

These unusual modes are in fact the first modes, that is, the modes with the longest wavelength in the structure (noting the discussion on modes in (Supplementary Note 2). Usually, such first modes of a potential are extended modes with low Q-factors. The fact that even the first modes in our structure are localized is another consequence of the interplay between evanescence and disorder. To show that these modes are indeed the first modes, we consider the log_10_ *I*(*z*,*λ*) (as in refs 32, 43), in a specific (arbitrarily chosen) realization, for incidence below and above the critical angle. Consider first the GHL case (*θ*=58°<*θ*_c_) shown in Fig. 5a. Here we see a relatively orderly array of wide modes and node lines (seen in Fig. 5a as thin bright curves). We denote the longest transmission resonance wavelength as *λ* and accordingly designate the mode associated with it (at the bottom of Fig. 5a) as the first mode. This is the wavelength above which disorder plays a role—for wavelengths much longer than *λ*, the field decays monotonically away from the interface, just as in the effective medium model (for *θ*>*θ*_c_). The resonance wavelengths for the sample simulated in Fig. 5a appear as dark horizontal lines, denoted by *λ*_2_,*λ*_3_ and so forth. Going back to the case at hand, we see that *λ*_1_∼5 μm—roughly equivalent to the sample size—and thus that *ξ*(*λ*_1_) is comparable to or is larger than the sample size. Owing to the logarithmic representation of Fig. 5b, the lines of minimum intensity are clearly visible. These lines, which we refer to as node lines, extend upwards from every mode in Fig. 5. If we follow the node line, which starts at the first mode, we see that this line intersects the second mode of the structure at *λ*_2_∼2.5 μm. This intersection is the location of the second mode's node. In a similar manner, the third mode is intersected by two such node lines originating from the first and second modes, and so forth. Eventually, for sufficiently short wavelengths, we do see spectrally narrow localized modes, but these modes are of high order and intersect multiple node lines. This behaviour, diffusive for long wavelengths and localized for short wavelengths, is broadly characteristic of the crossover from diffusive to localized waves. In our case, it characterizes the wavelength dependence in most of the GHL regime, but changes dramatically above *θ*_c_.

Next, we compare the first modes in the GHL regime at *θ*=58°<*θ*_c_ (Fig. 5a) to the first modes in the EME regime at *θ*=62°>*θ*_c_ (Fig. 5b). The landscape of resonant modes and node lines is visibly different in the EME regime. The modes in the EME regime are now much sharper, indicating that larger Q-factors are involved (since the integral of the intensity over space and frequency is expected to be the same in all modes). In particular, we see that the first mode is strongly localized (usually, with a Q-factor larger than that of the second and third modes). The crucial difference between Fig. 5a,b is the wavelength scale—for instance, the first mode in Fig. 5b is associated with *λ*_1_∼1 μm, which is much shorter than the size of the multilayer. Consequently, the mode associated with *λ* is characterized by a localization length also shorter than the multilayer size and is strongly localized. Being the first mode, it does not intersect any node lines, giving it the characteristic node-less shape seen in the modes in Fig. 4b. We emphasize that, owing to the logarithmic scaling, the node lines can be traced through the structure. Therefore, the mode we designated as the ‘first’ is truly the longest wavelength mode of the structure. The fact that the Anderson localization is so strong that it ‘overrides’ evanescence is in itself evidence of the potency of localization in this regime.

To demonstrate the generality of these results, we examine an ensemble of realizations of the disorder at *θ*=58° and 62° for structures of thickness *L*=6, 9 and 12 μm. The probability distribution of *λ* is plotted in Fig. 5c. For incidence at *θ*<*θ*_c_, the *λ*s are generally of the same order as *L*. The first mode in those cases leaks out of the disordered sample easily and has relatively low Q-factors. In contrast, for *θ*>*θ*_c_ the distribution of *λ* is centred at much shorter wavelengths and does not change appreciably as *L* is increased. Accordingly, for these modes, localization is of short range and we find Q-factors in the range of ∼10^2^–10^6^ (compared with Q∼10–10^3^ found for 58°). Moreover, these Q-factors are also much higher than the Q∼100 often reported in the literature for disordered multilayers^32^. For more information, consider the Q-factor probability density plot in Supplementary Fig. 13.

## Discussion

In conclusion, we have examined the two unique regimes of transport in which waves become sensitive to deep subwavelength features in the structure because of the vital role of evanescence. We showed transport is dominated by disorder in layers which are *d*∼*λ*/1,000 in thickness, far thinner than what is usually considered to be ‘deep subwavelength’. Moreover, localization in this regime is unusually potent, occurring on a length scale of *ξ*∼6*λ* and yielding significant Q-factors. This unique sensitivity to extreme subwavelength details suggests a number of potential applications, such as for spectral filtering or for sensing of miniscule features. Most importantly, suitable targets may already be found in the natural materials or biological samples^44^. However, this work focuses on the fundamental aspects—understanding the nature of wave transport in the presence of evanescent waves and the similarities and differences borne to conventional Anderson localization. Our results shed new light on localization phenomena, on the fundamental process of evanescent decay and on the interplay between these. Specifically, the transmission of an evanescent wave can be enhanced by the presence of subwavelength disorder and localized modes^45^,^46^. The competition between evanescence and disorder also leads to the formation of unique node-less localized modes. Finally, since the mechanism for phase accumulation in such structures is fundamentally different from the usual regime of localization, which involves propagating waves only, we believe that the interplay between evanescence and disorder will continue to generate intriguing questions, surprising answers and rich new physics.

### Data availability

The data that support the findings of this study are available from the corresponding author upon request.

## Additional information

**How to cite this article:** Herzig Sheinfux, H. *et al*. Interplay between evanescence and disorder in deep subwavelength photonic structures. *Nat. Commun.* **7**, 12927 doi: 10.1038/ncomms12927 (2016).

## Supplementary Material

Supplementary Information Supplementary Figures 1-13, Supplementary Notes 1-6 and Supplementary References
