# Author Correction: Interplay between evanescence and disorder in deep subwavelength photonic structures

**DOI:** 10.1038/ncomms16181

**Published:** 2018-02-08

**Authors:** Hanan Herzig Sheinfux, Ido Kaminer, Azriel Z. Genack, Mordechai Segev

Nature Communications 7: Article number: 12927; DOI: 10.1038/ncomms12927 (2016); Published: 10 06 2016; Updated: 02 08 2018.

The original version of this Article contained an error in the caption to Fig. 2b, which incorrectly read ‘Localization length as a function of *θ*, the angle of incidence for *λ*=1 μm’. The correct version indicates that the calculation was performed for a wavelength of ‘*λ*=0.5 μm’ and not ‘*λ*=1 μm’. This has now been corrected in both the PDF and HTML versions of the article.

