# Supplementary material for: Interplay between evanescence and disorder in deep subwavelength photonic structures
Source: Nat Commun. 2016 Oct 6;7:12927. doi: 10.1038/ncomms12927 (PMC5059687; doi:10.1038/ncomms12927)
Supplement: Supplementary Information — Supplementary Figures 1-13, Supplementary Notes 1-6 and Supplementary References [file ncomms12927-s1.pdf]

Supplementary Figures

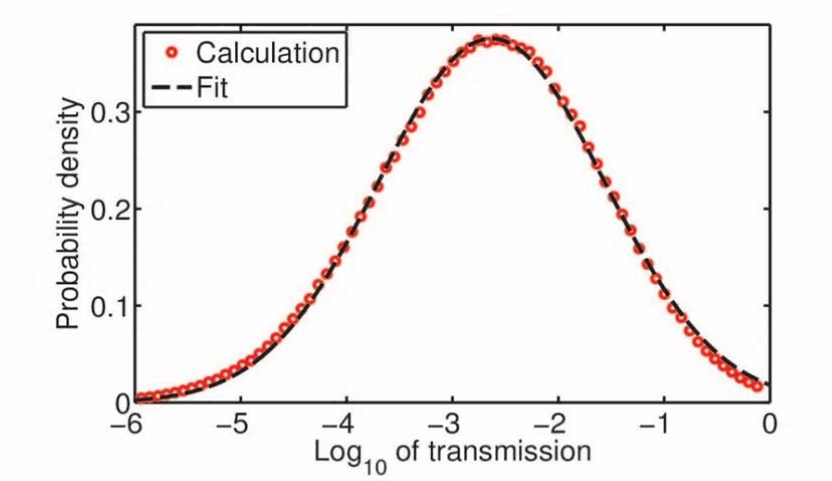

Supplementary Figure 1. Probability density of the log of transmission through an *N*=300 structure at $\theta = 60^{\circ}$ and $\lambda = 1\mu m$.

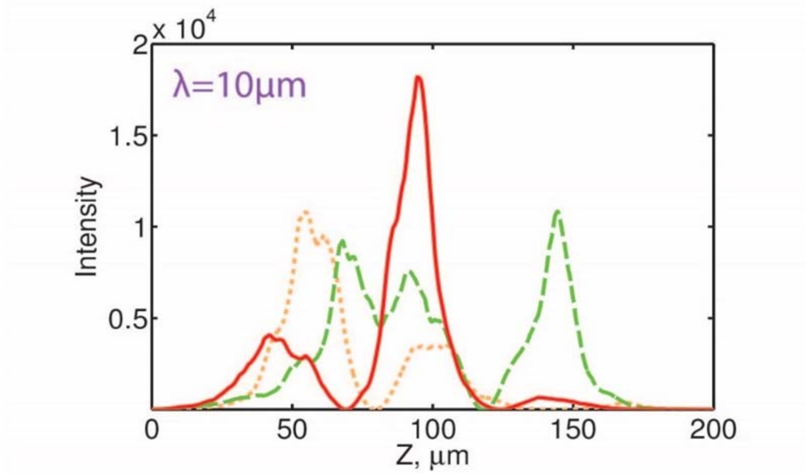

Supplementary Figure 2. Three localized modes for $\theta = 60^{\circ}$ and $\lambda = 10\,\mu m$. These modes are found by examining various realizations and finding the three "strongest" ones which have the highest transmission (out of an ensemble of 10,000 realizations). We sho the calculated intensity distribution of those realizations, with the intensity normalized relative to incident intensity.

Supplementary Figure 3. **(a-j)** In all of the subfigures below, the left hand side shows the intensity profile of the light for an incoming Gaussian beam incident at the critical angle, while the right hand side shows a longitudinal cross-section of the intensity of the trapped component inside the multilayer normalized to the peak intensity. Red represents the maximum intensity and green the minimum. The boundaries of this structure and the scale are the same as in Figs. 1b,c of the main text. The scale-bar in **a** applies to all figures below.

a.

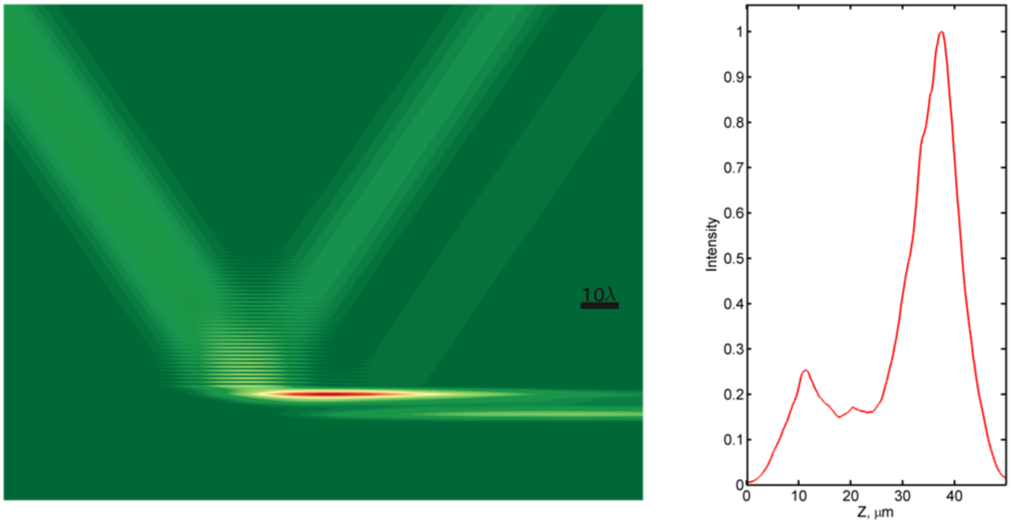

b.

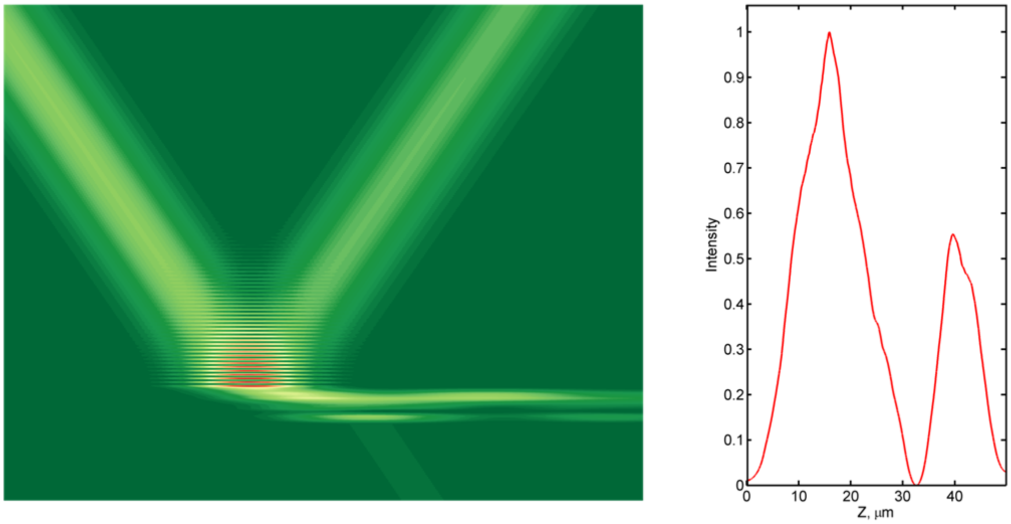

c.

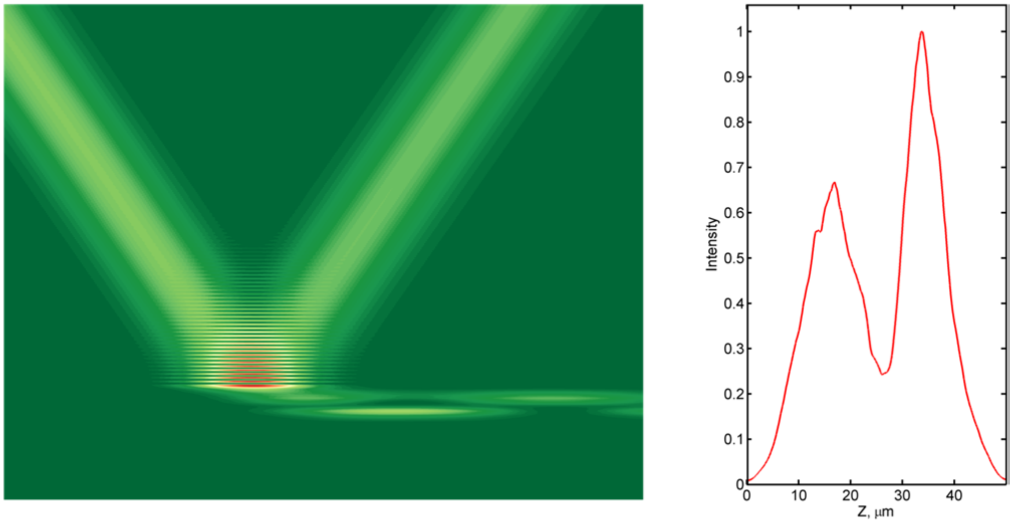

d.

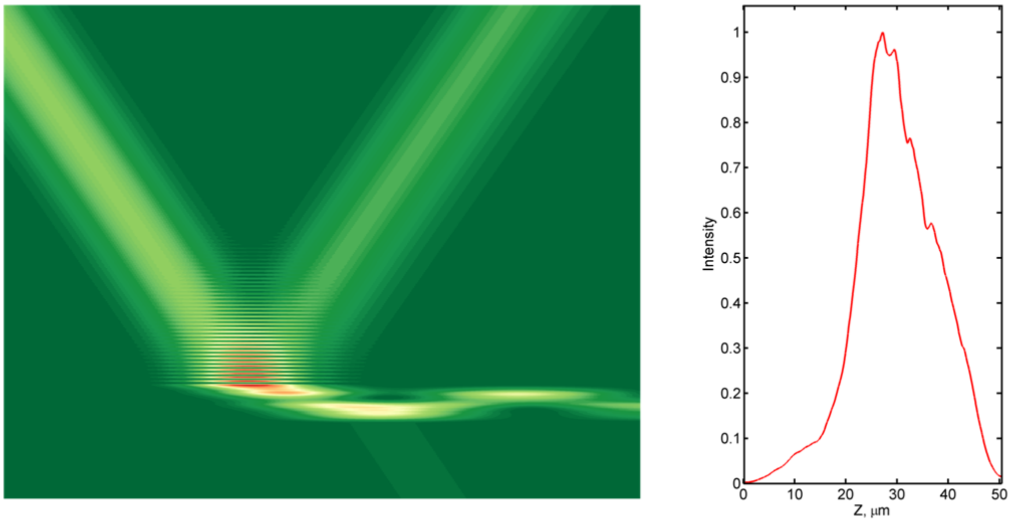

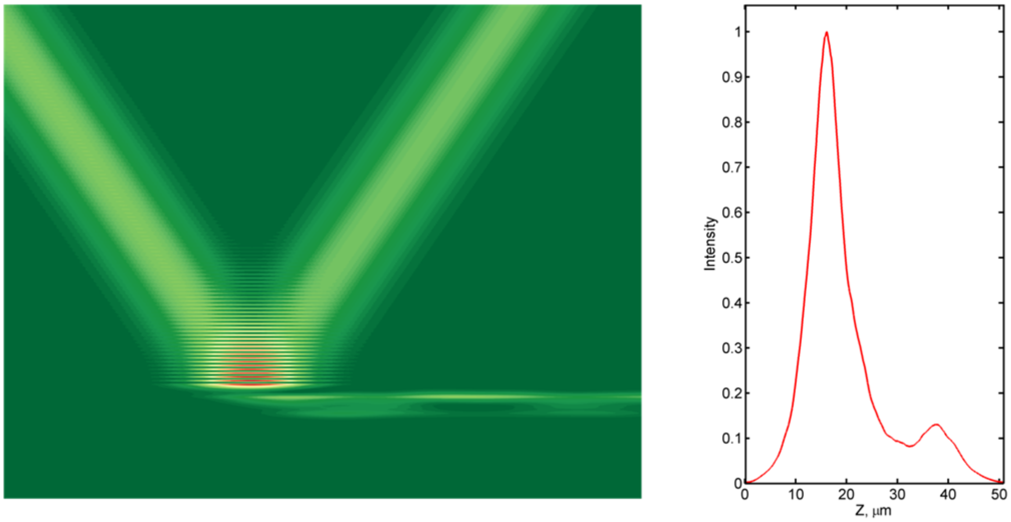
Intensity
Z, μm
0 0.1 0.2 0.3 0.4 0.5 0.6 0.7 0.8 0.9 1
0 10 20 30 40 50

e. 31

f. 32

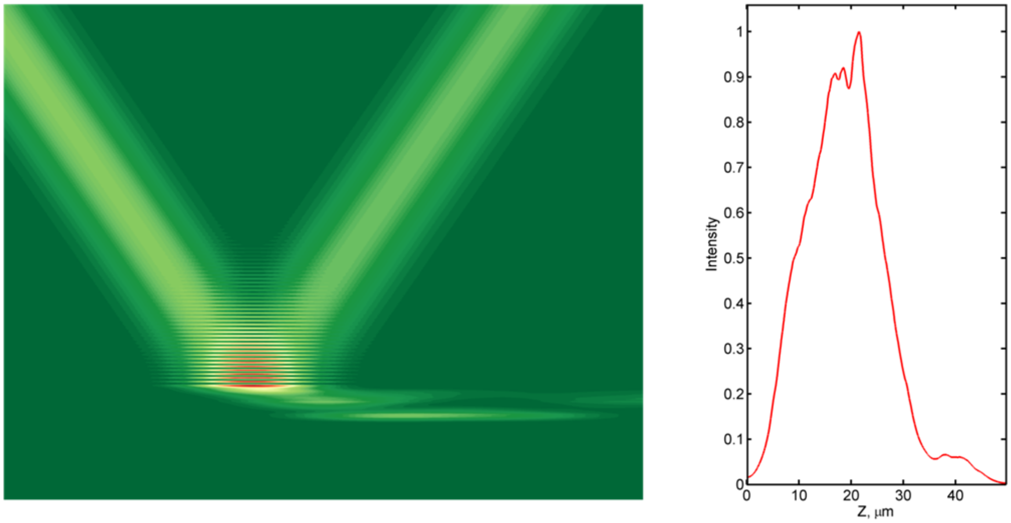
Intensity
Z, μm
0 0.1 0.2 0.3 0.4 0.5 0.6 0.7 0.8 0.9 1
0 10 20 30 40

g. 33

h. 34

i. 35

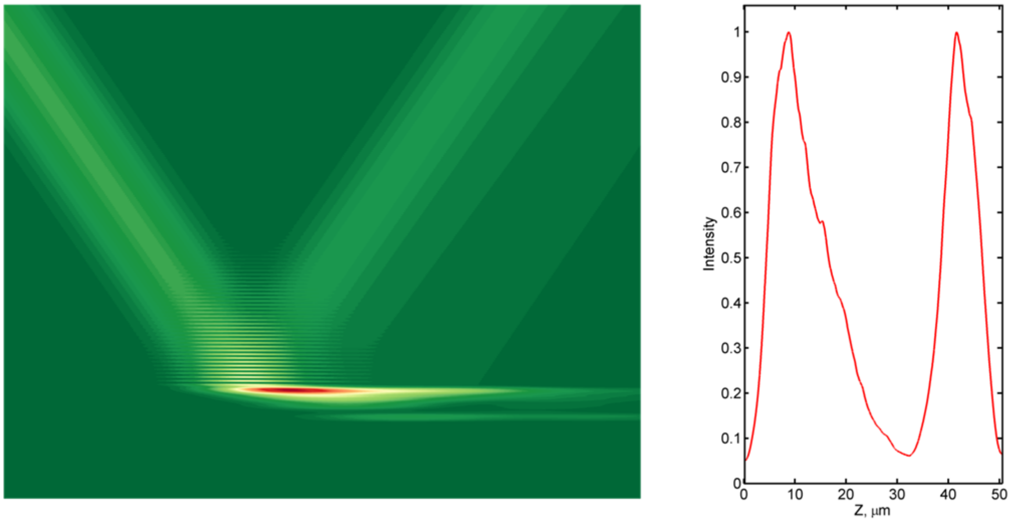

Intensity
Z, μm
0
0.1
0.2
0.3
0.4
0.5
0.6
0.7
0.8
0.9
1
0
10
20
30
40
50

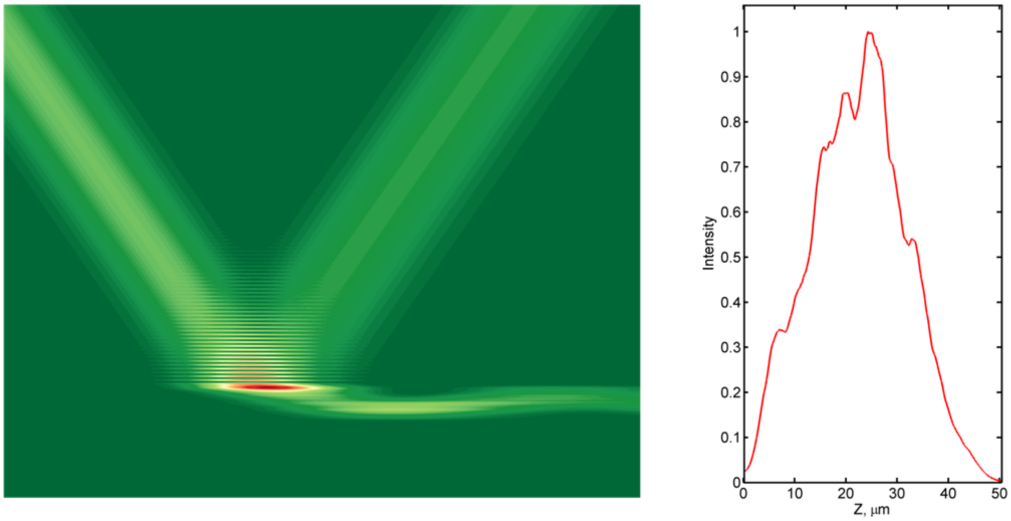

Intensity
Z, μm
0
0.1
0.2
0.3
0.4
0.5
0.6
0.7
0.8
0.9
1
0
10
20
30
40
50

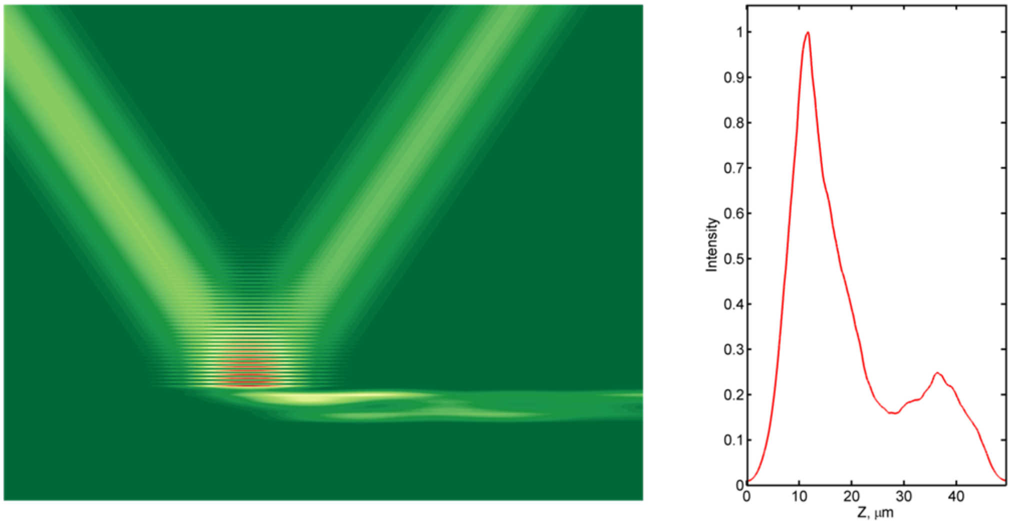

j. 36

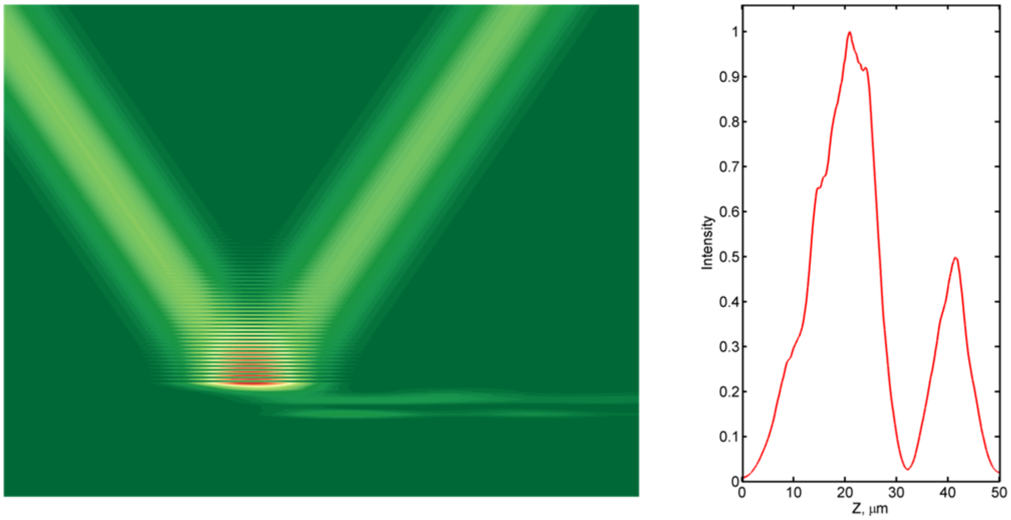

37

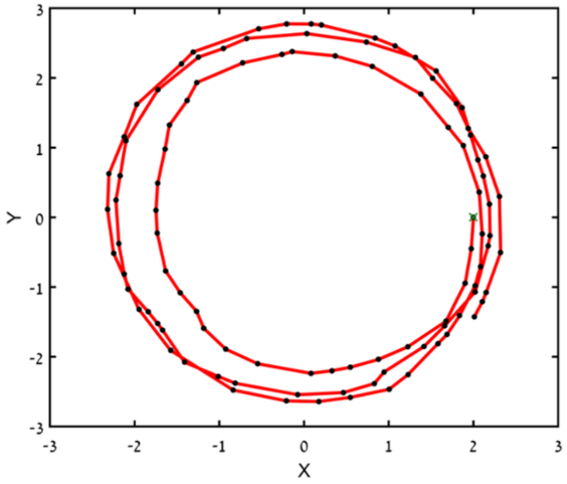

38

Supplementary Figure 4. Trajectory in the $X-Y$ plane for a disorder sample with $N=100$ layer pairs, at $\theta=20^{\circ}$, $\lambda=1\,\mu m$, $d=10\,nm$ (and all of the other parameters set as in the main paper). The black points are the values of X_m, Y_m, the green marker shows X_1, Y_1 and the red line guides the eye and connects subsequent dots.

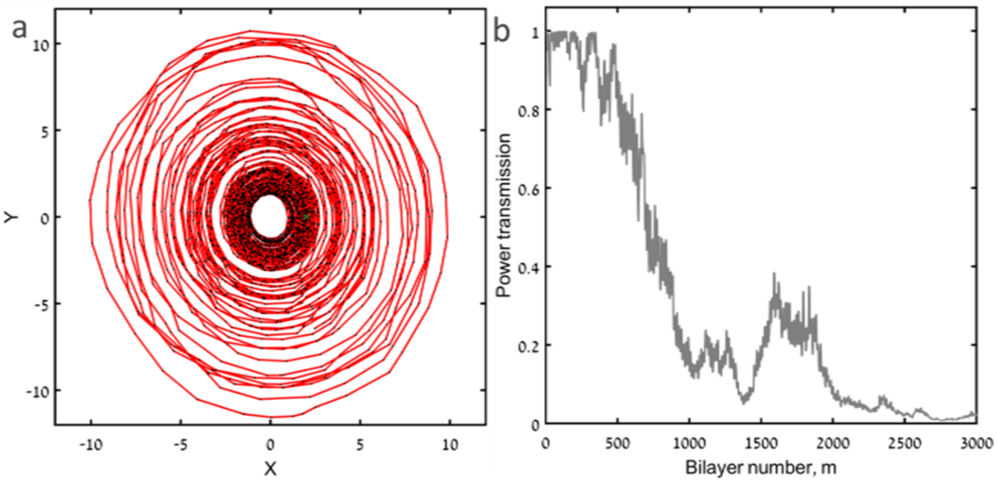

Supplementary Figure 5. **(a.)** Trajectory in the $X-Y$ plane for $N=3000$ layer pairs and $\theta=20^{\circ}$. **(b.)** Power transmission as a function of the number of layers in the multilayer for the same disordered realization.

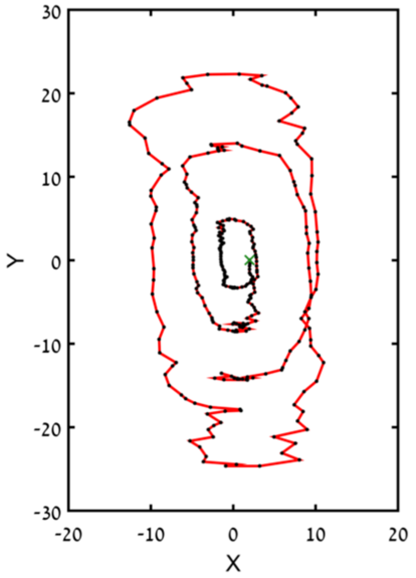

Supplementary Figure 6. A typical trajectory, for $N=300$ and $\theta=55^{\circ}$.

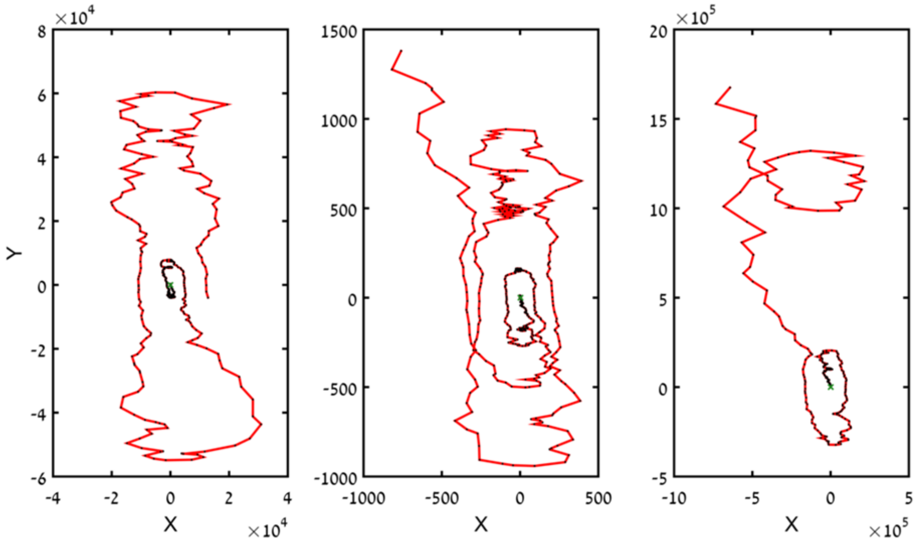

Supplementary Figure 7. Three typical trajectories, for $N = 600$ and $\theta = \theta_c = 60^\circ$. Note that the degree of localization (value of the maximum X and Y) differs in the different realizations, but also that the ratio of the X and Y maxima is similar.

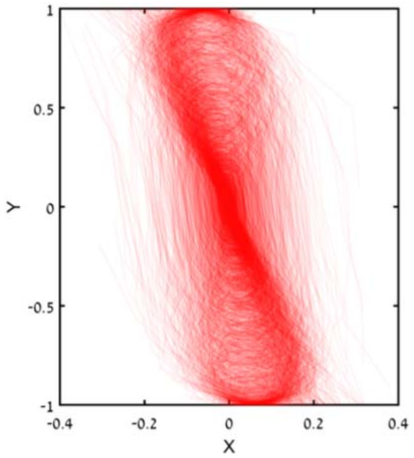

Supplementary Figure 8. The trajectories in 1000 realizations of disorder plotted over one another for $N = 3000$ and $\theta = \theta_c = 60^\circ$.

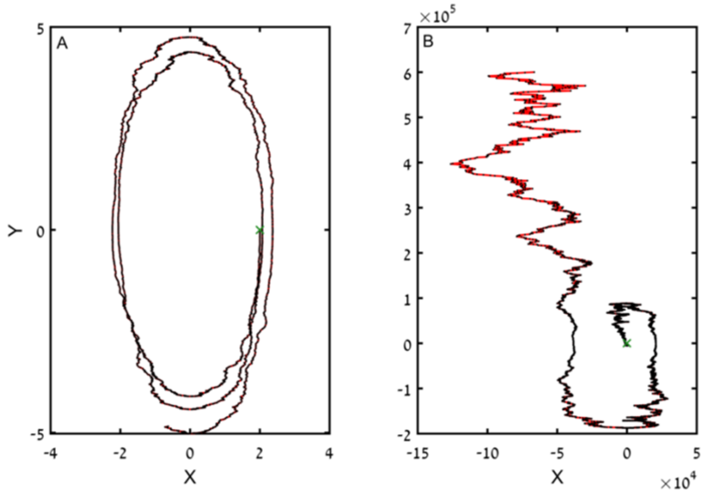

Supplementary Figure 9. **(a)** Typical trajectory in the $X-Y$ plane, for $N=2000$, $\theta=55^{\circ}$ and $\lambda=10\mu m$. **(b)** Trajectory for $N=10^{4}$ and $\theta=60^{\circ}$.

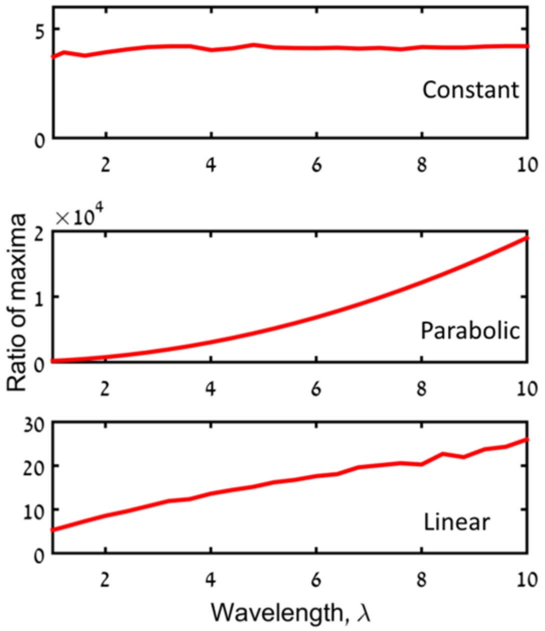

Supplementary Figure 10. The variation of the average ratio of maxima of X and Y with the wavelength in an ensemble of 1000 realizations. The ratio is calculated after propagation through a large number of layers, large enough that $Nd \gg \xi(\lambda)$. **(a)** for near critical incidence, $\theta=55^{\circ}$, **(b)** for critical incidence (at $\theta_c=60^{\circ}$) in a periodic disorder-free structure, **(c)** for critical incidence to a disordered structure.

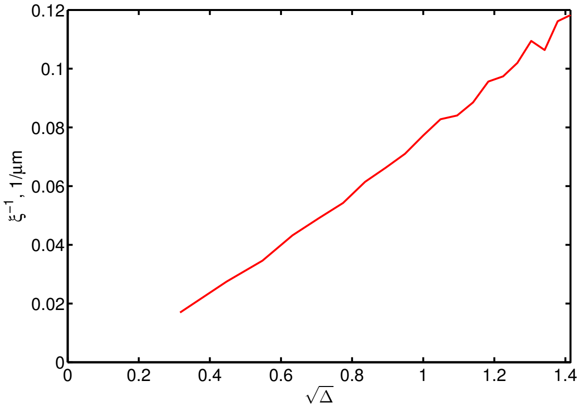

Supplementary Figure 11. Inverse localization length ($1/\xi$) as a function of $\sqrt{\Delta}$, showing a roughly linear dependence. In this figure we use the same parameters as Fig. 2a of the main text and $\lambda = 1\mu m$.

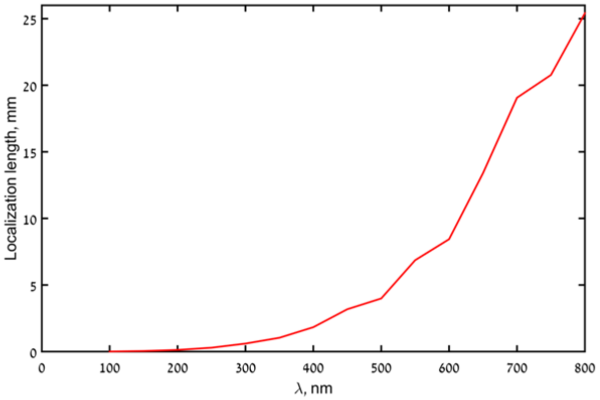

Supplementary Figure 12. Localization length as a function of wavelength, for correlated disorder with $d_{2n} = d_{2n+1} = random$. Note that the localization length scale is in mm and becomes so long that it takes prohibitively long times to continue the calculation to longer wavelengths. For the same reason, each point is the average over only 30 realizations. These calculations display a good fit to a λ^4 dependence.

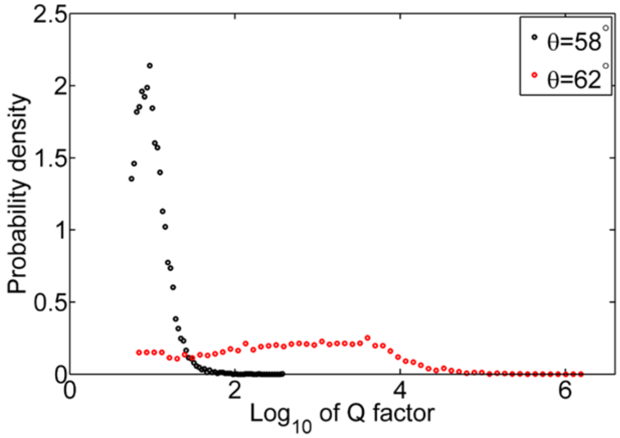

Supplementary Figure 13. Probability density of the 1st mode's Q-factor for two angles of incidence $\theta = 58°$ and $\theta = 62°$ below and above critical incidence. Results were extracted by taking distribution of the maximum intensity in an ensemble of 10^4 realizations of disorder and neglecting harder-to-identify diffusive modes with a Q-factor below 5.

Supplementary Note 1. The Abbe Transfer matrix formalism

The main tool in our calculations is the Abbe[1] transfer matrix formalism. This formalism is described in many places, but for completeness, we describe the Abbe formalism here and in the notations used throughout the article.

We consider a multilayer structure composed of alternating dielectric layers, each layer with uniform permeability and a permittivity. The dielectric constant just beyond the input and output of the structure have arbitrary dielectric constants

$$
\begin{aligned}
&\varepsilon(z<0)=\varepsilon_{in} \\
&\varepsilon\left(\sum_{1}^{n-1} d_n < z < \sum_{1}^{n} d_n\right)=\varepsilon_n \text{ ,[1]} \\
&\varepsilon\left(\sum_{1}^{N} d_n d < z\right)=\varepsilon_{out}
\end{aligned}
$$

for n=0,1,2,…, 2N-1 where $\varepsilon_n = \varepsilon_a$ for even values of n and $\varepsilon_n = \varepsilon_b$ for odd values of n. Consider now the decomposition of the (TE polarized) electromagnetic field into forward and backward propagating waves $E_{tot}(z)=(E_+ + E_-)\hat{y}$ where $E_+(nd < z < (n+1)d) = U_n e^{ik_{z,n}(z-nd)-i\omega t}$, $E_-(nd < z < (n+1)d) = V_n e^{-ik_{z,n}(z-nd)-i\omega t}$ and $k_{z,n} = k_0\sqrt{\varepsilon_n - \varepsilon_{in}\sin^2(\theta)}$, where θ is the angle defined in the text (see Fig. 1 of the main text). U_n, V_n are complex constants indicating the amplitude and phase of the wave immediately after the interface. Outside the structure, we assume the wave propagates (is not evanescent), so that the propagation constants $k_{z,0}$ and $k_{z,n}$ $k_{z,n}$ $k_{z,N}$ are real.

The transfer matrix relating the field in two adjacent layers is[2,3]:

$$
\begin{pmatrix} U_n \\ V_n \end{pmatrix} = T_{n,n+1}\begin{pmatrix} U_{n+1} \\ V_{n+1} \end{pmatrix} = \frac{1}{t_{n,n+1}}\begin{pmatrix} exp(-ik_{z,n}d) & r_{n,n+1}exp(-ik_{z,n}d) \\ r_{n,n+1}exp(+ik_{z,n}d) & exp(+ik_{z,n}d) \end{pmatrix}\begin{pmatrix} U_{n+1} \\ V_{n+1} \end{pmatrix} \quad [2] .
$$

The transfer matrix for a stack of such layers, extending from the nth to the mth surface (with $n < m$), is therefore:

$$\begin{pmatrix} U_n \\ V_n \end{pmatrix} = T_{n,m} \begin{pmatrix} U_m \\ V_m \end{pmatrix} = \begin{pmatrix} A_{m,n} & B_{m,n} \\ C_{m,\mathrm{n}} & D_{m,n} \end{pmatrix} \begin{pmatrix} U_m \\ V_m \end{pmatrix} = \prod_{k=n}^{m} T_{k,k+1} \begin{pmatrix} U_m \\ V_m \end{pmatrix} \quad [3] \, .$$

After calculating the relevant transfer matrices, the transmission coefficient for a wave incident on the nth layer is $1/A_{m,n}$ and the reflection coefficient is $B_{m,n}/A_{m,n}$.

Given the set of transfer matrices and assuming the illumination is incident from the left ($U_0 = 1, V_N = 0$), we find $\begin{pmatrix} U_n \\ V_n \end{pmatrix}$ inside a structure with N layers ($n < N$):

$$\begin{pmatrix} U_n \\ V_n \end{pmatrix} = \frac{1}{A_{0,n}A_{\mathrm{n,N}} + B_{0,n}C_{n,N}} \begin{pmatrix} A_{n,N} \\ C_{n,N} \end{pmatrix} [4] \, .$$

Repeating this process for various angles of incidence to give the different plane wave components of an incident Gaussian wave, we find the field distribution for an incident beam.

This is how all the results presented in this article are calculated, including all the figures. The results of these calculations are also corroborated by scattering matrix calculations. While both scattering and transfer formalisms are analytically identical, the scattering matrix calculations are known to be more stable numerically in systems with evanescent waves. However, for the case at hand, we find practically identical results in both formalisms.

Supplementary Note 2. Characterization of the GHL regime

Both evanescence and localization lead to exponential decay of the fields transmitted through a multilayer stack. How then can localization be definitively demonstrated in a medium in which the wave is evanescent in some parts of the sample? This can be done by showing unique signatures of localized waves are present in an ensemble of disordered samples. Here we consider three such characteristics: (1) the variation of the scaling of transmission upon the strength of disorder, (2) the equality of the variance of the logarithm of transmission and the absolute value of the average of the logarithm of transmission, as required by the single parameter scaling hypothesis, ad (3) the presence of spatially localized modes. These characteristics have been demonstrated in the main text. Here we specifically contrast these properties to those of typical systems supporting evanescent waves.

1. **Scaling of transmission.** In the Goos-Hanchen localization regime (see definition of GHL in the main text), we find that, without disorder, i.e, in a perfectly periodic multilayer, the transmission and intensity inside the structure is a periodic function of the thickness of the overall structure, which resembles the transmission through a Fabry-Perot cavity. Therefore, full transmission can be obtained even through very thick structures[4]. The exponential decay in the (average) transmission of a random ensemble, as seen in Figs. 2a,b of the main text, only appears when disorder is added. Moreover, the exponential decay of transmission depends strongly on the degree of randomness of the layer thicknesses. For example, drawing the layer thicknesses from a rectangular distribution from the 8 to 12 nm range (instead of the 2 to 18 nm range used all throughout the article), leads to a substantial increase in the localization length ξ and weakens all reported localization effects.

2. **Statistics of transmission.** As shown in Supplementary Figure 1a, below, the statistical distribution of the transmission through the multilayer sample is log-normal, which is the typical distribution for localized waves in 1D disordered systems[5,6]. In addition, the variance of the log of transmission is essentially equal to the magnitude of the average of the log of transmission. This feature is in accord with the single-parameter scaling (SPS) hypothesis, which is a known

characteristic of Anderson localization in 1D[6,7]. Specifically, we find that $\mathrm{var}\left(\ln T\right) \approx 1.05L/\xi$ or better throughout the GHL regime.

3. Modal makeup of field. The field inside the sample does not decay monotonically, as occurs for evanescent waves. Instead, the intensity profile of the EM field can exhibit one or more peaks inside the sample, with intensities much higher than the input intensity. In particular, for realizations associated with high transmission, such as those shown in Supplementary Figure 1b, relatively high Q-factors are seen (even for $d \sim \lambda/1000$). The intensity profiles in Supplementary Figure 2 are the modes of the system. The frequency dependence of these modes is found to be Lorentzian, and the typical length scale of the modes is comparable to the value of ξ found in the paper (for example, in Fig. 2a). These features are all characteristic of Anderson localized systems (for example, see [8,9]). The spacing between the modes is generally greater than the width of these modes (see for example Fig 5b and the top of Fig 5a), which complies with the Thouless criterion for localization[9,10].

Note that here and in the main text, we refer to quasi-normal modes as 'modes'. Quasi-normal modes are the resonances of an open system and exhibit a Lorentzian linewidth. When referring in the main text to the “first mode”, we mean the quasi-normal mode with the lowest frequency possible for a specific angle of incidence. For a potential of finite height and support, such a first mode is always expected to exist. It is essential distinguish between quasi-normal transmission modes and the transverse modes of a waveguide, which are a completely different entity, related to the waveguide profile.

Supplementary Note 3. Localization of beams: additional examples

In any physical experiment, the incident beam is a wave packet of plane waves which carries finite power, unlike idealized plane waves. Supplementary Figure 3, shows 10 examples of a 2D Gaussian beam incident at the critical angle for different random realizations of disorder in the structure, all with the same parameters as the structure as in Fig. 1c.

Due to random nature of localization, there is a significant difference between the manifestation of localization in different disordered realizations. But notably, all of these examples have much in common. Mainly, they all show a very strong decay in transmission. The exact amount of transmitted light, however, changes drastically, where some examples (sub figure **i**) show no transmission and some (subfigure **b** and **d**) show a small amount of light can get through. In addition, in most of the figures there is a visible trapping effect, induced by a localized mode (as in subfigure **a** and **f**), but some figures (as in subfigure **i**) show almost no transverse propagation of trapped energy.

Supplementary Note 4. Analytic derivation of the scaling relation of the localization length

Introduction and definitions

Our goal here is to show that, in the vicinity of the critical angle for TIR, the localization length scales with the wavelength as $\xi \sim \lambda$ for asymptotically long wavelengths and therefore that localization remains wavelength scaled in deep subwavelength structures. The technique relies on the Hamiltonian mapping method[11], where we map the problem of transmission through a stack of layers of random thicknesses to a trajectory in the plane of $X = E_y$, the transverse electric field, and $Y = -ik_z^{-1}B_z$, the longitudinal component of the magnetic field. Without disorder, these trajectories are known to be simple ellipses. Under the influence of weak disorder, the elliptical trajectories are perturbed and acquire multiple "kicks" from the disorder. Each kick can change the radius of the trajectory in a random manner, either increasing it or decreasing it, but on average the effect of disorder accumulates, manifesting itself in the exponential decay of the field, indicating that the wave is localized due to the disorder.

In the analytic treatment which follows, the matrix formalism we use differs from the Abbe formalism described above. In the formalism used here, we use the X ,Y basis instead of the U,V basis (of forward and backward waves). Otherwise, we use the same parameters and notation as elsewhere in the text. Specifically, we assume that $d = d_a = d_b$, that the range of variations in d is comparable to d , and that $d << \lambda$.

Since the field in the entire bilayer in deep subwavelength structures is almost constant, it is sufficient to take only the field value at a single point at the beginning of the high-permittivity layer in each bilayer X_m, Y_m. This point is arbitrarily chosen to be the beginning (leftmost point) of the high-permittivity layer. We further assume that the odd layer in the mth bilayer, where $n = 2m-1$, are high permittivity layers, in which the waves propagate with $k_H \in \mathrm{R}$ and that the even layer in the each bilayer ($n = 2m-2$) has low permittivity with $k_L \in i\mathrm{R}$. We only consider waves incident exactly at the critical angle, $\theta = \theta_c$ and TE polarization. From the definition of θ_c, we

have $k_{z,\mathrm{eff}} = \sqrt{\bar{\varepsilon} k_0^2 - k_x^2} = 0$ and therefore $k_{z,H} = \sqrt{\varepsilon_H k_0^2 - k_x^2} = \sqrt{\left(\varepsilon_H - \bar{\varepsilon}\right) k_0^2}$ $= -i\sqrt{\left(\varepsilon_L - \bar{\varepsilon}\right) k_0^2} = -ik_{z,L}$. A discussion of the behavior for other angles of incidence appears in the main paper. As a result, the ratio of the layer impedances is $Z_a Z_b^{-1} = i$. We then have, $\varphi = \varphi_a = -i\varphi_b = k_{z,H} d$. We emphasize that the matrix solution is an exact solution of Maxwell's equations.

Following the conventions of the transfer matrix formalism, we do not calculate the field transmitted through the structure, but rather the field amplitude required at the input of the structure to obtain a field with unit amplitude at the output. Therefore, larger radii of a trajectory imply that the field decays significantly before reaching a (lower) value at the input. Thus, proving that the wave is localized entails showing that the trajectories diverge. Proving that this localization is due to disorder and not simple evanescence is addressed above in Supplementary Note 2. This convention has a significant added benefit of simplifying the interpretation of visualizations of calculated trajectories.

Using this notation, the field amplitudes in the nth bilayer are related to the amplitude of the adjacent cell by

$$X_{m+1} = \tilde{A}_m X_m + \tilde{B}_m Y_m \qquad Y_{m+1} = -\tilde{C}_m X_m + \tilde{D}_m Y_m \quad [5].$$

The factors $\tilde{A}_m$, $\tilde{B}_m, \tilde{C}_m, \tilde{D}_m$ are the transfer matrix coefficients for the bilayer structure[38]. By taking the lowest orders in $\frac{d}{\lambda}$ of these coefficients, we obtain

$$\begin{aligned}
\tilde{A}_m &= 1 + \varphi^2 - \frac{1}{2}\ \eta_H^2(m) - \frac{1}{2}\ \eta_L^2(m) + i\ \eta_H(m)\eta_L(m) \\
\tilde{B}_m &= -\frac{1}{3}\varphi^2 + \eta_H(m) - i\eta_L(m) \\
\tilde{C}_m &= 2\varphi + \eta_H(m) + i\eta_L(m) \\
\tilde{D}_m &= 1 - \varphi^2 - \frac{1}{2}\ \eta_H^2(m) - \frac{1}{2}\ \eta_H^2(m) - i\ \eta_L(m)\eta_L(m),
\end{aligned} \qquad [6]$$

where $\eta_j(m) = k_j\left(d_{jm} - d\right)$, with d_{Hm}, d_{Lm} are widths of the high and low permittivity layers at period of index m .

Unperturbed periodic system

As a constructive example, we begin with the disorder-free case, where we perform the following canonical transformation:

$$\begin{aligned} X_m &= v^{-1}Q_m\cos\tau - vP_m\sin\tau \qquad & Q_m &= vX_m\cos\tau + vY_m\sin\tau \\ Y_m &= v^{-1}Q_m\sin\tau + vP_m\cos\tau \qquad & P_m &= -v^{-1}X_m\cos\tau + v^{-1}Y_m\sin\tau. \end{aligned} \quad [7]$$

The new coordinates, Q_m, P_m, occupy a circular trajectory, instead of the elliptical trajectory of X_m, Y_m. We require that Q, P rotate at a fixed angular frequency $\gamma = \frac{1}{2}\varphi^2$. This value can be found by substituting in the expression for $\tilde{A}_m$, $\tilde{B}_m, \tilde{C}_m, \tilde{D}_m$ and following the resulting algebra, as in[38]. Note that for calculating γ, higher order terms in φ must be considered, but not elsewhere in the derivation. More simply, we can use the following dispersion relation, whose validity for describing the bulk behavior in our case is shown in[4]

$$\cos\gamma = \cos\varphi_a\cos\varphi_b - \tfrac{1}{2}\left(Z_aZ_b^{-1} + Z_bZ_a^{-1}\right)\sin\varphi_a\sin\varphi_b \ [8].$$

With Z_a (Z_b) the wave impedance in the a (b) layer. Here, γ, the phase accumulation across a single pair of layers, is related to the effective medium wavenumber via $k_{z,eff} = \gamma / d$. Usually, we expect to that $\gamma \sim \varphi$, but at critical incidence, the linear term exactly cancels since $Z_aZ_b^{-1} = -Z_bZ_a^{-1}$ and we find $\gamma = \frac{1}{2}\varphi^2$ for $\lambda \gg d$.

To solve for Q_m, P_m and find v, τ, we note that the factors A_m, B_m, C_m, D_m are greatly simplified for the disorder-free case, with $\eta_L(m) = \eta_H(m) = 0$:

$$\begin{aligned} A_m &= 1+\varphi^2 \qquad & B_m &= -\frac{1}{3}\varphi^2 \\ C_m &= 2\varphi \qquad & D_m &= 1-\varphi^2. \end{aligned} \quad [9]$$

To find the values of v, τ, we calculate

$$\begin{aligned} Q_{m+1} &= \left(A_m \cos^2\tau + B_m \cos\tau\sin\tau - C_m \cos\tau\sin\tau + D_m \sin^2\tau\right)Q_m \\ &+ v^2\left(-A_m \cos\tau\sin\tau + B_m \cos^2\tau + C_m \sin^2\tau + D_m \cos\tau\sin\tau\right)P_m. \end{aligned} \quad [10]$$

In order to obtain rotation with an angular frequency γ, we require

$$\begin{aligned} &A_m \cos^2\tau + B_m \cos\tau\sin\tau - C_m \cos\tau\sin\tau + D_m \sin^2\tau = \cos\gamma \\ &-A_m \cos\tau\sin\tau + B_m \cos^2\tau + C_m \sin^2\tau + D_m \cos\tau\sin\tau = v^{-2}\sin\gamma, \end{aligned} \quad [11]$$

giving

$$2\varphi \sin^2\tau = v^2 \sin\gamma \, [12].$$

Similarly,

$$\begin{aligned} P_{m+1} &= \left(-A_m \cos\tau\sin\tau + B_m \sin^2\tau - C_m \cos^2\tau + D_m \cos\tau\sin\tau\right)Q_m \\ &+ v^{-2}\left(A_m \sin^2\tau - B_m \cos\tau\sin\tau - C_m \cos\tau\sin\tau - D_m \cos^2\tau\right)P_m \,, \end{aligned} \quad [13]$$

giving

$$2\varphi \cos^2\tau = v^2 \sin\gamma \; [14].$$

Thus,

$$\tau = \frac{\varphi^2}{4}, \qquad v^2 = \frac{2}{\varphi} \; [15].$$

Having found the parameters of the unperturbed trajectory, we would proceed to calculate the effect of disorder, in a manner similar to [11]. However, this calculation yields the unphysical result of a localization length which is independent of the wavelength for asymptotically large wavelengths, because the disorder-coupled magnetic field is much larger than the magnetic field existing in the absence of disorder. The calculation above shows that in the absence of disorder B_z is of order φ^2, which is a manifestation of the wavelength dependence of the effective medium

permittivity, which converges as $\Delta(\varphi)=\varepsilon_{\text{eff}}-\bar{\varepsilon}\sim\varphi^2$ (see discussion in [4]). On the other hand, the disorder provides coupling of the electric field to the magnetic field which is of order φ (through the $v^2\tilde{B}_m\cos^2\tau$ term). Therefore, to properly treat the magnetic field in the presence of disorder we suggest to change the unperturbed state and take as an ansatz a different basic state for which B_z is of order φ. In what follows we justify this ansatz further, by numerical evidence.

Numerical investigation of the trajectories

The trajectories in the $X-Y$ plane can be calculated numerically, providing a vivid image of the system's behavior. We start with the case of near-normal incidence, where we expect a perturbed-elliptical trajectory which spirals away due to disorder. Note that in all the figures below, we draw only the real part of the fields. For the parameters here, each circuit is nearly circular. The same simulation implemented on a larger multilayer shows the trajectory spiraling out considerably. This corresponds to the decay of transmission and to localization.

For $\theta = 55^{\circ}$, closer to critical incidence, the trajectory becomes less regular and more stretched. The impact of disorder in this case is seen to be stronger, inducing larger random jumps in the trajectory.

At the critical angle, the effect of disorder becomes dominant and the trajectories are more complex and random. The jumps in the trajectory are larger and the trajectories include loops or wild zig zags or may rapidly fly away. Nevertheless, for sufficiently large N, the trajectory always revolves around the center of the pattern and eventually forms a highly distorted and elongated spiral pattern. The ratio of the maxima of X and Y in all trajectories of sufficient length is, on average, roughly the same.

Unlike the cases of lower angles of incidence, the effect of disorder here is dramatic and different realizations of disorder produce different and highly distorted trajectories. The resemblance to an elliptical trajectory is also much weaker. However, normalizing all all of trajectories in an ensemble to their maximum radius and drawing them one on top of the other produces a picture that is easier to interpret visually.

When the wavelength is increased asymptotically, one might expect the trajectory to become closer and closer to the unperturbed elliptical trajectory. Indeed, for most angles of incidence, this is precisely what happens. However at the critical angle, the effect of disorder is not diminished as the wavelength increases. For $\lambda = 10^4 d$, the trajectories remain as distorted in exceedingly long wavelengths. The number of

bilayers, N, required for the trajectory to evolve does increase, but it does not converge to the unperturbed trajectory.

For example, Supplementary Figure 9b shows a trajectory that is qualitatively similar to the ones in Supplementary Figure 7, except for the larger *ratio* of the maxima of X and Y. To determine the rate of elongation in the disordered system, we can calculate the ensemble average of this ratio as a function of the wavelength.

Away from the critical angle, the ratio of the maxima of X and Y is almost independent of the wavelength (Supplementary Figure 10a). At critical incidence, this ratio is wavelength-dependent, but as our earlier analysis suggested - this dependence is not parabolic as in periodic structures (Supplementary Figure 10b). In Supplementary Figure 10c, this ratio still increases in a disordered system at critical incidence, but at a much slower, roughly linear, rate. Such a linear dependence in the wavelength complies with our intuition that the size of B_z should be dominated by the disorder-induced coupling coefficient, which was shown earlier to be linearly related to the wavelength.

Scaling of the localization length

In light of the numerical analysis above, we suggest the following ansatz – the basic trajectory appears to be an ellipse with $v^4 \sim \varphi^{-1}$ (instead of $v^4 \sim \varphi^{-2}$ found for the unperturbed trajectory). There is a strong intuitive argument for this assumption: the strong coupling of Y to X inflates the trajectory and effectively prevents the magnitude of B_z from being as low as predicted by effective medium theory. At the same time, it should also be stressed that this assumption is only expected to produce the dependence in φ and therefore the scaling of the localization length, but not the exact dependence or other features of the system.

With this assumption, we can now demonstrate that the localization length scales linearly with the wavelength, as we have found numerically in the main text. To do so, we find recurrence relations for the radius, defined as, $R_m^2 = Q_m^2 + P_m^2$. Keeping only the lowest order terms in φ, and using $v^2 \sim \sqrt{\varphi}$, as per our assumption, we obtain

$$Q_{m+1} = Q_m + 2\varphi^{-\frac{1}{2}}\left(\eta_H(m) + i\eta_L(m)\right)P_m \, [16],$$

$$P_{m+1} = P_m - 2\varphi^{\frac{1}{2}}\left(2\varphi + \eta_H(m) - i\eta_L(m)\right) [17].$$

Thus,

$$R_{m+1}^2 = R_m^2 + 4\varphi^{-\frac{1}{2}}\left(\eta_H(m) + i\eta_L(m)\right)Q_m P_m + 4\varphi^{-1}\left(\eta_H(m) + i\eta_L(m)\right)^2 P_m^2 \, [18].$$

We denote $\sigma_H^2 = \left\langle \eta_H^2(m)_m \right\rangle$, the variance of d_n in the high permittivity layers and $\sigma_L^2 = \left\langle \eta_L^2(m)_m \right\rangle$ for the low permittivity layers. We assume that no correlation exist, so that $\left\langle \eta_H(m)\eta_L(m) \right\rangle = 0$ as stated earlier. We then average over n, using $\left\langle \eta_H(m) \right\rangle = \left\langle \eta_L(m) \right\rangle = 0$ to get

$$\frac{R_{m+1}^2}{R_m^2} = \varphi 4\sigma^2 \cos^2\theta_m \, [19],$$

where $\tan\theta_m = Q_m P_m^{-1}$ and $\sigma^2 = \sigma_H^2 + \sigma_L^2$. Here, we neglect correlation terms such as $\eta_H(n)\sin\theta_m$, since, for long wavelengths, θ_m changes slowly (i.e., over a large number of layers), whereas η_H changes rapidly and averages to zero in the absence of long-range correlation in d_n. Since $\cos^2\theta_n$ is generally a factor of order unity, we neglect it for the purposes of scaling and are left with the relation

$$\xi = \frac{2d}{ln\frac{R_{m+1}^2}{R_m^2}} \sim \frac{1}{k_H\sigma^2} \sim \frac{\lambda}{\sqrt{\Delta}} \text{[20]},$$

where $\Delta = \varepsilon_H - \varepsilon_{\text{eff}} = \frac{1}{2}(\varepsilon_H - \varepsilon_L)$.

The linear scaling of ξ with the wavelength, which is what we find here, is one of the main findings of this work. It corresponds to the localization length remaining short, typically a few wavelengths, even for exceedingly long wavelengths, as demonstrated numerically in Fig. 2a in of the main text. In a similar vein, the scaling with Δ can also be verified numerically and goes as indicated in Supplementary Equation 11. It is interesting to note that it differs from the $\Delta^{-\frac{3}{2}}$ dependence predicted theoretically in [21] for a related scenario.

Supplementary Note 5. Effect of interbilayer correlations on wavelength scaling

In the analysis above, we assumed that η_H, η_L are uncorrelated random variables. However, considering the derivation above, we see that if $|\eta_H| = |\eta_L|$ at every bilayer then the v^1 term of $\tilde{B}_n$ cancels. Accordingly, in such a case, the scaling we find no longer holds and we find instead a $\xi \sim (\lambda / d)^4$ dependence, as demonstrated the figure below. We do not derive this alternative scaling relation explicitly here – the derivation and its result for this case are very similar to those in [38]. However, the contrast between the correlated case and the uncorrelated cases is exceedingly large. For example, two structures which differ only on a deep subwavelength (say, nanometric) level correlations can have an entirely different optical response, either reflecting or transmitting light completely, with localization lengths that differ by many orders of magnitude This is another example to the sensitivity of this regime to subwavelength details, where certain correlated random stacks exhibit complete delocalization and other correlations exhibit complete localization.

Supplementary Note 6. Q-factor distribution for the first mode

In Fig. 6c of the main text, we show the wavelength at which the first mode of a structure is obtained. We can also find the Q-factor distribution of these modes for two angles of incidence, one below and one above the critical angle. These Q-factors are important for potential applications, and can also help us to better understand the transverse dynamics in Figs. 1b,c, since they indicate the transverse localization length.

These Q-factors are important for the analysis in the main text for several reasons. First, because they demonstrate the strength of the localization relative to normal incidence localization. Q-factors reach 10^6 which is remarkably high for Anderson localization, especially for thin samples. Moreover, the presence of high-Q modes, facilitates the enhanced transmission effects we describe in the main text, as well as the wide spread in the transverse direction of a finite beam. A better understanding the Q-factor distribution is required to understand these effects.

References

[1] F. Abeles, La théorie générale des couches minces, *J. Phys. Radium*, **11**, 307–310 (1950).

[2] Katsidis, C. C. & Siapkas, D. I. General transfer-matrix method for optical multilayer systems with coherent, partially coherent, and incoherent interference. *Appl. Opt.* **41,** 3978-3987 (2002).

[3] Heavens, O.S. *Optical Properties of Thin Solid Films.* (Dover Publications, New York, USA, 1991).

[4] Herzig Sheinfux, H., Kaminer, I, Plotnik, Y., Bartal, G. and Segev, M. Subwavelength multilayer dielectrics: ultrasensitive transmission and breakdown of effective-medium theory. *Phys. Rev. Lett.* **113,** 243901 (2014).

[5] Lagendijk, A., van Tiggelen, B. and Wiersma, D. S. Fifty years of Anderson localization. *Phys. Today* **62,** 24-29 (2009).

[6] Anderson, P. W., Thouless, D., Abrahams, E. and Fisher, D. New method for a scaling theory of localization. *Phys. Rev. B* **22**, 3519-3526 (1980).

[7] Deych, L., Lisyansky, A., and Altshuler, B. L. Single-parameter scaling in one-dimensional Anderson localization: exact analytical solution. *Phys. Rev. B* **64,** 224202 (2001).

[8] Bertolotti, J., Gottardo, S., Wiersma, D., Ghulinyan, M. and Pavesi, L. Optical necklace states in Anderson localized 1D systems. *Phys. Rev. Lett.* **94,** 113903 (2005).

[9] J. Wang, A. Z. Genack, Transport through modes in random media. Nature. **471**, 345–8 (2011).

[10] Sebbah, P., Hu, B., Klosner, J., and Genack, A. Z. Extended quasimodes within nominally localized random waveguides. *Phys. Rev. Lett.* **96,** 183902 (2006).

[11] Izrailev, F. & Makarov, N. Localization in correlated bilayer structures: from photonic crystals to metamaterials and semiconductor superlattices. *Phys. Rev. Lett.* **102**, 203901–1 (2009).
